# Surgical smoke: a matter of hygiene, toxicology, and occupational health

**DOI:** 10.3205/dgkh000469

**Published:** 2024-03-05

**Authors:** Nurettin Kahramansoy

**Affiliations:** 1 Department of Surgery, İzmir Bozyaka Education and Research Hospital, İzmir, Turkiye

**Keywords:** surgical smoke, diathermy, toxic exposure, pathogen transmission, occupational risk, prevention, control

## Abstract

The use of devices for tissue dissection and hemostasis during surgery is almost unavoidable. Electrically powered devices such as electrocautery, ultrasonic and laser units produce surgical smoke containing more than a thousand different products of combustion. These include large amounts of carcinogenic, mutagenic and potentially teratogenic noxae. The smoke contains particles that range widely in size, even as small as 0.007 µm. Most of the particles (90%) in electrocautery smoke are ≤6.27 µm in size, but surgical masks cannot filter particles smaller than 5 µm. In this situation, 95% of the smoke particles which pass through the mask reach deep into the respiratory tract and frequently cause various symptoms, such as headache, dizziness, nausea, eye and respiratory tract irritation, weakness, and abdominal pain in the acute period. The smoke can transport bacteria and viruses that are mostly between 0.02 µm and 3 µm in size and there is a risk of contamination. Among these viruses, SARS-CoV-2, influenza virus, HIV, HPV, HBV must be considered. The smoke may also carry malignant cells. The long-term effects of the surgical smoke are always ignored, because causality can hardly be clarified in individual cases.

The quantity of the smoke changes with the technique of the surgeon, the room ventilation system, the characteristics of the power device used, the energy level at which it is set, and the characteristics of the tissue processed. The surgical team is highly exposed to the smoke, with the surgeon experiencing the highest exposure. However, the severity of exposure differs according to certain factors, e.g., ventilation by laminar or turbulent mixed airflow or smoke evacuation system. In any case, the surgical smoke must be removed from the operation area. The most effective method is to collect the smoke from the source through an aspiration system and to evacuate it outside. Awareness and legal regulations in terms of hygiene, toxicology, as well as occupational health and safety should increase.

## Abstract (de)

Der Einsatz von Devices zur Gewebedissektion und Blutstillung während des Eingriffs ist im Allgemeinen unumgänglich. Elektrisch betriebene Devices wie Elektrokauter, Ultraschall- und Lasergeräte erzeugen chirurgischen Rauch, der mehr als tausend verschiedene Verbrennungsprodukte enthält. Darunter befinden sich große Mengen an krebserregenden, mutagenen und potentiell teratogene Noxen. Der Rauch enthält Partikel in einem weiten Größenspektrum, das bis zu 0,007 µm betragen kann. Die meisten Partikel (90%) im Elektrokauterisationsrauch sind ≤6,27 µm groß. Chirurgische Masken können Partikel <5 µm nicht filtern. Daher gelangen 95% der durch die Maske gelangenden der Rauchpartikel tief in die Atemwege und verursachen in der akuten Phase häufig Symptome wie Kopfschmerz, Schwindel, Übelkeit, Reizungen der Augen und Atemwege, Schwäche und Bauchschmerzen. Der Rauch kann Bakterien und Viren transportieren, die meist zwischen 0,02 µm und 3 µm groß sind, so dass ein Kontaminationsrisiko besteht. Unter diesen Viren sind SARS-CoV-2, Influenzaviren, HIV, HPV und HBV zu nennen. Der Rauch kann auch Krebszellen enthalten. Langfristige Auswirkungen des OP-Rauchs werden stets ignoriert, da die Kausalität im Einzelfall kaum geklärt werden kann.

Die Menge des Rauchs hängt von der Technik des Chirurgen, dem Belüftungssystem des Operationsraums, den Eigenschaften des verwendeten Devices, der eingestellten Energiestufe und den Eigenschaften des verarbeiteten Gewebes ab. Das Operationsteam ist in hohem Maße dem Rauch ausgesetzt, am stärksten jedoch der Chirurg. Das Ausmaß der Exposition hängt jedoch von verschiedenen Faktoren ab, insbesondere von der Art der Belüftung (laminare Luftströmung oder turbulente Mischströmung) und dem Rauchabzugssystem. In jedem Fall muss der chirurgische Rauch aus dem Operationsbereich entfernt werden. Die wirksamste Methode besteht darin, den Rauch durch ein Absaugsystem an der Quelle zu erfassen und ins Freie abzuführen.

Abschließend ist festzustellen, dass das Bewusstsein und die gesetzlichen Vorschriften in Bezug auf Hygiene, Toxikologie, Arbeitsschutz und Sicherheit verbessert werden sollten.

## Introduction

When the author of this article complained to the manager who formerly was an active surgeon that surgical smoke emitted during surgery clinically affected him, causing symptoms, the manager (inactive surgeon) replied: *‘sometimes I miss the smell of that smoke*’. His lack of awareness of the danger of the smoke prompted the author to review this topic. Such lack of awareness lays the foundation for a process that can ultimately lead to various symptoms and diseases. The fact that some characteristics of surgical smoke are little-known or insufficiently understood seems to be the most important barrier to awareness. Therefore, the aim of this study was to review the current knowledge about surgical smoke. 

We know that using electrically powered devices for tissue dissection and hemostasis is almost unavoidable during surgical procedures. These include electrocautery devices, ultrasonic scalpels, and laser ablation units. These devices generate by-products which are mostly termed “surgical smoke”. However, terms such as plume, aerosol and vapor are also used. The latter (vapor, aerosol and plume) are mostly preferred for larger particles suspended in the air and are produced by processes other than combustion [1], [2].

## Method

The PubMed database of the US National Library of Medicine was searched for the MeSH terms ‘surgical’ and ‘smoke’, and the term ‘surgical smoke’ with other terms such as ‘plume, vapor, exposure, characteristics, particulate matter, ventilation, and operating room’. English-language literature was checked by title and abstract for eligibility according to the aim of this study. Then full texts of potentially eligible studies were assessed. A number of the most frequently cited review studies were used as a guide to the original reports. To obtain the most recent knowledge, articles published within the last five years were selected; however, earlier articles with quantitative analyses were not neglected.

## Contents of surgical smoke

### Chemical combustion products

Most of the substances in surgical smoke have been known for many years [1], [3], [4], [5], [6]. In the last decade, 150 types of chemical compounds were mentioned, but today 1,064 types of volatile organic compounds (VOCs) are reported. It is not difficult to foresee that this number will increase with technological development [7], [8].

The main components found in surgical smoke are CO_2_ (44.4%), water vapor (31.8%), 2-propenal (syn. acrolein; 10.7%), and propane (8.3%) (Table 1 (Tab. 1)) [9]. Some compounds (methanol, methane, ethane, ethylene, acetone, formaldehyde, 1,3-butadiene, hydrogen cyanide, benzene, methylpropene, toluene) are also detected in large quantities [6], [8], [10], [11], [12], [13], [14]. Aldehyde derivatives (e.g., formaldehyde), toluene, 1–2 dichloroethane, benzene and acrylonitrile are among the compounds of greatest concern [4], [6], [15]. Formaldehyde, benzene, and 1,3-butadiene (carcinogenic group 1), propane, ethylene (and derivates in carcinogenic groups 1 to 3), 2-propenal (acrolein), styrene, methane, and ethane (and derivates in carcinogenic group 2A), ethylbenzene, methylpropene, 1,2-dichloroethane, toluene, and acrylonitrile (carcinogenic group 2B), as well as heptane and vinyl acetylene are known not only to be carcinogenic but also mutagenic [9], [12], [13], [14], [16], [17], [18].

### Particles

In addition to chemical compounds, the smoke contains cells, cell particles, erythrocytes and hemoglobin [19], [20]. The particles exist in different sizes and amounts that vary greatly depending on the method used for the tissue ablation and dissection. Most particles created by electrocautery are as small as 0.007 µm in size; while the ultrasonic scalpel leads to the formation of larger particles [3], [16], [19], [21], [22], [23], [24], [25]. The size of particles produced by the laser are detected range more widely, from very small to large sizes [7], [26]. Electrocautery also creates smaller particles in higher quantity and concentration, in addition to large particles with high total weight [23] (Table 2 (Tab. 2)). It is easy for the small particles [particulate matter (PM) <2.5 µm (PM_2.5_)] to remain suspended in the air for a time, while the suspension of PM >10 µm does not last long.

Malignant cells have been identified among the suspended particles and cells [27]. Due to their size, bacteria (2–3 µm), viruses (0.02–0.2 µm), and proteins (≤0.001 µm) can also be found suspended in surgical smoke [3], [28], [29]. Essentially, transmission of bacteria by the smoke and their infectivity have been demonstrated experimentally and clinically. For example, inoculation of the smoke into bacterial culture showed that 5 of 13 smoke samples contained coagulase-negative staphylococci [30]. In an experimental spine surgery study, bacteria were detected in 95% of swabs from a smoke sample, and one or more bacteria consistent with tissue swabs grew in 84% of these samples [31]. 

The presence and transport of viruses or chromosomal particles in surgical smoke has been investigated for quite some time. Viruses that have high risk of pathogenicity and are most emphasized in terms of transmission are hepatitis B virus (HBV) (0.045 µm), human papillomavirus (HPV), human immunodeficiency virus (HIV) (0.01–0.13 µm), and SARS-CoV-2 (0.125 µm) [29], [32]. HBV was detected by PCR in surgical smoke in 10 of 11 HBV-positive patients [33]. Moreover, in these cases, closed surgical methods such as laparoscopic or robotic surgery, which reduce the amount of smoke released into the environment, were used [33]. 

HPV DNA was detected in the surgical smoke of 2 of 7 cases treated with a CO_2_ laser [34]. The reason that HPV DNA was not detected in more smoke samples may be the long smoke-collection tube, to the interior of which the virus or its DNA probably adhere. In a study of 24 patients with high-grade squamous intraepithelial lesions of the cervix who underwent excision with loop electrocautery, the smoke contained HPV in 4 patients [35]. HPV subtype was the same in the tissue and in the smoke sample. Only in one experimental study was coronavirus RNA detected in surgical smoke proportionate to the viral load of the tissue (1/10^6^–1/10^5^), but the virus was reported to be inactive [36]. There is just one publication on HIV; it stated that HIV DNA could not be detected in the vaporous debris. However, wash culture of the tube through which the plume was transported revealed the presence of p24 HIV gag antigen at the end of the first week in 3 of the 12 samples and at the end of the 14^th^ day in 1 of the 12 samples [37]. This result indicates that the HIV virus is transported by surgical smoke.

### Factors influencing surgical smoke amount and composition

The amount of smoke varies greatly, depending on many factors (Table 3 (Tab. 3)). By employing a conservative approach and technique for tissue dissection and hemostasis, the frequency of power-device use can be limited, thus limiting the amount of smoke. Table 2 (Tab. 2) shows that different power devices produce particles in different amounts and concentrations. 

Electrocautery devices produce higher smoke concentrations and smaller particles, 90% of which are ≤6.27 µm in size [24]. Monopolar electrocautery devices produce the highest amount and concentration of particles [19], [21], [22], [25], [38]. The smoke concentration emitted by the monopolar device is 82 and 721 times higher than that of bipolar and ultrasonic devices, respectively [22]. The amount of particles smaller than 2.5 µm (PM <2.5) produced by monopolar electrocautery is quite high (up to 2,258 µg/m^3^) [39], [40], [41]. The average size of the particles in laser smoke is 0.07 µm, and 77% are <1.1 µm [3]. Bipolar electrocautery and ultrasonic devices produce less smoke [22], [25], [41]. 

Another factor affecting the amount of particles is the energy level of the device used. That is, as the energy level increases, more smoke (especially PM <2.5) is produced, and the amount of particles increases with the extension of the diathermy period [10], [26], [42], [43]. Other factors that determine the amount of smoke and particle size are the characteristics of the tissue processed. More smoke and smaller particles are generated during the dissection and hemostasis of dense tissues such as liver, kidney and muscle [9], [21], [23], [24]. These situations vary even within a tissue itself. For example, different amounts of smoke, particles and chemical compounds are released from the different tissue components (subcutaneous tissue, fat, mammary gland and breast-tumor tissues) during breast surgery [10]. The general opinion is that many factors affect the amount and content of surgical smoke; however, this is mentioned as a limitation of some studies [44].

## Factors affecting the exposure of the surgical team to smoke

There are several factors – other than those limiting smoke formation – which influence the exposure of the operating-theater staff (Table 4 (Tab. 4)), as explained below.

### Surgical methods

Significantly more fine (PM<2.5) and large particles are present in the smoke of open colorectal surgery compared to that of the laparoscopic method [41]. A study reports that the total amount of VOCs in the smoke produced in open surgery is slightly higher than the amount in the smoke from thoracoscopic surgery [8]. However, almost all of the selected VOCs (especially pentadiene, crotonaldehyde, g-butyrolactone) were found to be significantly higher in the smoke generated during open surgery [8]. Interestingly, higher concentrations of acetonitrile and acetaldehyde were measured during thoracoscopy [8]. Another study stated that a high amount of acetaldehyde, propionaldehyde and formaldehyde still existed in the smoke produced by laparoscopic surgery, even if the smoke was filtered. Furthermore, the amount of formaldehyde was reported to be above the determined upper limit of risk (0.016 ppm) [15]. Dense smoke (245.7 µg/m^3^) is generated during open surgery. However, if the smoke generated during laparoscopic surgery is discharged into the operating theater from the trocar valve, the smoke exits as a spray with high pressure and concentration (517.5 µg/m^3^), which actually increases the smoke exposure of the operating staff [39].

Mintz et al. [45] believed that the risk of SARS-CoV-2 transmission by smoke in laparoscopic surgery is less than that in open surgery. In contrast, others have suggested a return to open vs instead laparoscopic surgery during the COVID-19 pandemic, as there may be less risk of aerosols with faster operating times [46]. Up to now, no study has demonstrated the ability of the virus SARS-CoV-2 to be transmitted during a surgical procedure whether open or laparoscopic [47]. Even if laparoscopic surgery is associated with a lower risk of surgical-site infections, a shorted healing time and lower risk for incisional hernia, an advantage of open surgery may be the isobaric setting around the surgical field. However, during any use of electrocoagulation, tissue-specific aerosols may develop. Kwark’s study [33] found that surgical smoke contained HBV in 10 of 11 hepatitis B patients who underwent laparoscopic or robotic surgery. 

### Insufflation

Laparoscopic, minimally invasive techniques produce aerosols derived from the induced pneumoperitoneum. The risk of an aerosol carryover is reduced by insufflation-systems equipped with smoke-gas elimination (smoke evacuation) and defined CO_2_ feeding and discharge; therefore, these systems are to be preferred for patients with COVID-19 [46]. Alternatively, it is recommended that older insufflator-instruments are used which have disposable smoke gas-filters (acc. to ISO 29463) with a Luer-taper connection to remove smoke-gases by filtering [48].

### Trocar valve

Accordingly, evacuation of the contaminated smoke at high speed and concentration from the trocar valve into the operating theater may increase the risk of transmission compared to the open surgery.

### Proximity to the source of surgical smoke

The place where the smoke is most concentrated is undoubtedly its source. Smoke density attains a maximum in the area up to ~150 cm from the source of the smoke [49]. Therefore, the surgeon, who is closest to the smoke source is most exposed to smoke (3,000 µg/m^3^, 10^5^ particles/cm^3^) [9], [21], [38], [50], [51], followed by the residents and nurses around the operation table. While performing surgery on HPV-related cervical pathologies, gynecologists have an increased risk of HPV transmission they are close to the smoke source [52].

Although some publications claim exposure to surgical smoke is equal for all staff in the operating theater [8], [21], [53], the present author points out that those authors reached their conclusion based on the smoke-sample collection methods used during the studies (such as long smoke transfer lines) and the use of ventilation systems in the operating theater which aspirate from the ceiling (conventional) or blow from the ceiling (laminar flow) [21], [53]. For example, Kocher et al. [8] concluded that the surgeon and other operating theatre personnel had similar exposures, but they measured the average concentration of VOC to be the highest, at 272.69 parts per billion (ppb) per volume (max. 8,991 ppb), near the surgeon. The present author finds this conclusion controversial, because those authors evaluated only the distribution of the average maximum amount of the VOCs they selected, not the total distribution of all substances.

### Surgical masks

**Figure 1:**
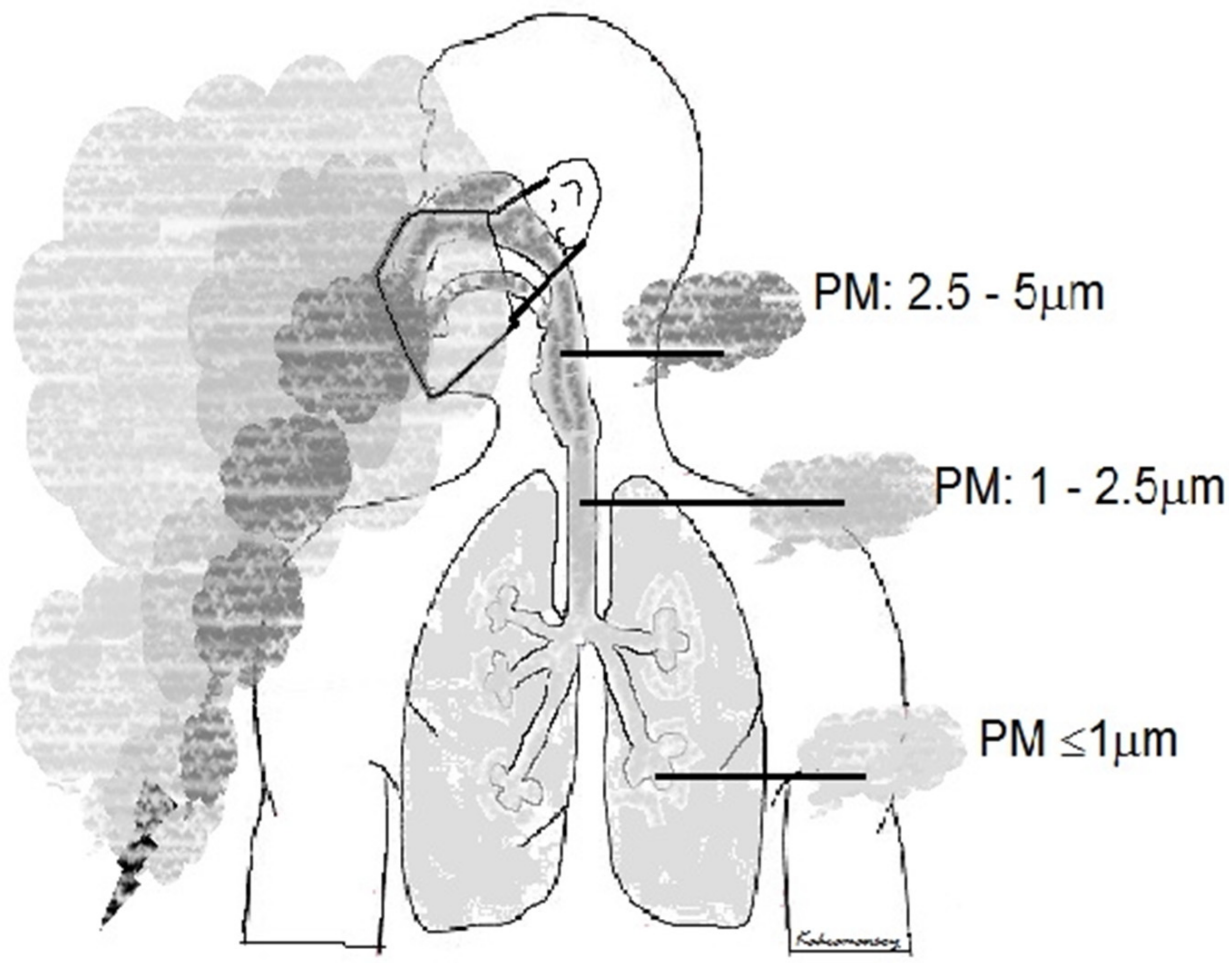
The sizes of the smoke particles passing through the surgical mask and the regions they reach in the respiratory tract (PM=size of particulate matter) (author’s drawing)

The characteristics of the mask used by the operating theater team affect the severity of exposure to smoke and the risk of possible microorganism transmission. Depending on the characteristics of the mask, 20% to 100% of particles smaller than 1 µm penetrate through it [54]. Mostly surgical masks are used during surgery. It is known that surgical masks cannot filter particles <5 µm. This means the smoke passes almost completely through the mask and reaches to the airways of the surgical team (Figure 1 (Fig. 1)) [28], [55], [56].

Additionally, the effect of the surgical mask is close to null because it is not properly tied during the operation and does not completely close the airways [54], [56], [57]. Thus, particles, most of which are smaller than 5 µm, can reach deep into the respiratory tract (Figure 1 (Fig. 1)) [28]. The limited effect of the surgical mask may be to stop substances and droplets larger than 5 µm. An experimental study reported that 99.80% of the ineffective viral RNA carried by surgical smoke is filtered out by the surgical mask, which clearly contradicts the above information [36]. In a study reporting that surgical masks reduced droplet-borne SARS-COV-2 transmission by 42%. This rate seems possible based merely on the droplet size being >5 µm [58]. N95 masks, defined as filtering face-piece (FFP) respirators, filter much smaller particles (at least 95% of 0.3 µm particles) but are not as effective (99%) as FFP3 and N100 masks [32], [55], [56]. The limitations of the use of N95 and N100 masks are that they fit tightly to the face, making breathing difficult, causing temporary facial damage, and are relatively expensive.

## Removing the surgical smoke from the breathing zone

### Removal from the source

Passive or active smoke-removal methods are available. Active evacuation of the smoke as soon as it emerges from its origin can be performed by a central aspiration system or a mobile device with a filter via the suction line located close to the smoke’s origin [50], [59]. In such a case, the total amount of VOCs is 3,000 µg/m^3^ (8,700 particles/cm^3^) around the operating table decreases to approximately 175 µg/m^3^ (1,600 particles/cm^3^) by using a smoke evacuation system (SES) [50], [59]. Some authors state that the use of SES does not significantly reduce the total amount of VOCs, but they did detect a significant decrease in some specific VOCs [8]. Those authors mentioned acrylonitrile, pentadiene, methyl-2-butenalin, hydrogen cyanide, formaldehyde, butadiene and butenes among these VOCs [8]. However, they did not evaluate the effect of the central ventilation system on the results of their study.

SES is effective if it is close to the source of the smoke. The power of the smoke evacuation system increases when it is placed closer to the smoke source and the power setting of the device is increased [42]. Many researchers have presented many inexpensive and alternative innovative methods for evacuation of smoke from near the source (since mobile smoke filter devices are expensive) [16], [60], [61], [62], [63], [64]. In addition, irrigation with water is recommended as an innovative technique, especially during laparoscopic laser application. Some authors reported a significant reduction in the amount of smoke by irrigating with water [65]. A different study recommended precipitating the smoke in the abdomen by an electrostatic method during laparoscopic surgery. Using the electrostatic method, there was no need to interrupt the surgical procedure, clean the camera lens, or additionally evacuate the abdominal air [66]. If the smoke precipitation in the abdomen is not performed, exposure to smoke increases indirectly.

### Smoke removal from the operating theater through a ventilation system

Central ventilation systems blow the smoke and diffuse it into the operating theater, then discharge it through the channels in the walls of the room. This could be suggested as a passive method for smoke removal. Conventional operating-room central ventilation systems are classified as laminar or turbulent mixed airflow systems and negative pressure systems [67]. The conventional system does not blow filtered air; however, it provides ambient air exchange by drawing air through the channels in the ceiling. The laminar flow ventilation system (LVS) blows the filtered air from the ceiling or walls and exchanges the ambient air by drawing it through channels in the walls. The negative pressure system provides suction with reinforced laminar flow from the ceiling and negative pressure from the walls in the closed operating theatre. This system was first used during the SARS epidemic in 2003 [67].

The characteristics of the operating-room central ventilation system produce different effects (independent of the smoke) [67], [68], [69], [70]. Conventional ventilation systems increase the amount of all particles near the surgeon and the risk of bacterial contamination in the operating area [69], [70]. This risk is also mentioned for LVS in two other studies, which allows anticipating a similar effect for the smoke [69], [70]. LVS, on the other hand, has been found to significantly reduce the quantity and concentration of particles near the surgeon [38], [49], [71]. It is thought that LVS achieves its reducing effect by first dispersing the dense smoke from the source through the operating room and then removing it through the air vents in the walls. Contrary to this, Hofer et al. [68] reported that LVS caused more smoke to concentrate near the surgeon. The authors suggested that surgical lamps caused smoke to concentrate near the surgeon by blocking the tabletop airflow and increasing the temperature below the lamps [68]. Other studies stated that LVS distributed the dense smoke from the source through the operating room, exposing the personnel far from the operating table to the same extent as the surgeon at the table [8], [53]. Van Gestel [53] asserted that the resident and nurse were more exposed to the smoke than was the surgeon. They performed this study in an operating room with LVS with horizontally placed blower and evacuation channels on the opposite walls [53]. This might have allowed the smoke to be directed towards the other members of the team.

The number and mobility of personnel in the operating theater generally disrupt the air flow that increases the number of macro- and microparticles in the air and indirectly causes surgical site infections [72], [73]. Thus, it can be predicted that the increase in the number and mobility of personnel will delay smoke evacuation by disrupting the airflow created by the central ventilation system. However, no research on this issue was found.

## Symptoms caused by surgical smoke in healthcare personnel

### Acute side effects and risks

In general, large particles are of biological concern due to the transport risk of cell and tissue particles as well as droplets, while small particles pose more of a chemical concern as they can reach as far as the alveoli (Figure 1 (Fig. 1)) [5], [20], [22], [28].

Particles smaller than 5.0 µm, which cross the surgical mask barrier and reach the alveoli, cause noticeable, acute effects. A number of them are dizziness-drowsiness (74.2%), headache (72.8%), cough (70.3%), dizziness (68.6%), tearing (65.7%), nausea (63.4%), throat irritation (56.6%), smell of smoke in the hair (43.7%), and fatigue (28.2%) (Table 5 (Tab. 5)) [74], [75], [76]. Probabilities of viral and bacterial transmissions are mentioned above.

### Chronic risks

In public life, prolonged exposure to small particles suspended in the air has been associated with increased risk of lung, breast, uterine, ovarian, colon, and prostate cancers [77]. Surgical smoke has been reported to cause cardiovascular diseases, lung injuries, such as pneumonia, chronic bronchiolitis, emphysema, and pulmonary fibrosis, in addition to blood disorders (e.g. anemia, leukemia), in the long-term [3], [4], [5], [20], [78]. Besides to the carcinogenic, mutagenic and potential teratogenic risks of it, smoke is also known as a tumor-cell carrier, and tumor cells can be cultivated from smoke samples [27], [79]. The mutagenic effect of surgical smoke is estimated to be greater than that of cigarette smoke, and may increase depending on the type of tissue processed. It was found that the mutagenic potential of the smoke resulting from the ablation of 1 g of tissue with electrocautery was equivalent to the smoke of 6 unfiltered cigarettes. Accordingly, it is predicted that in the operating theater, a surgeon exposed to surgical smoke is subject to the same mutagenic power created by the smoke of 27–30 unfiltered cigarettes per day (depending on the brand) [4]. Some authors have stated that the concentration of small particles in the operating theater does not exceed that of office air but is much lower than in cigarette smoke, and that the mutagenic potential of the surgical smoke is negligible [43]. They speculated that this was probably due to the intermittent use of electrocautery and high-velocity ventilation during surgery.

## Awareness

It is a rare surgeon who does not use a power device. However, the rate of the evacuation of the smoke from the operation table ranges from 14% to 70% [78], [80]. If evacuation is needed, the majority (90%) of the surgeons use the standard central aspiration system for this purpose [78]. According to a 2012 study, only 66% of the operating theaters had an SES [4]. However, the current rate of SES equipment in the operating theaters may have increased by now. Elsewhere, the SES usage rate is reported to be as low as 16.8% to 0% [74], [78], [81]. The low usage rate of SES varied according to the surgeon and the surgical procedure performed. The reasons why the surgeons used SES were to obtain clear vision (76.9%), safety (61.5%) and deodorization (15.3%) [4]. 

Awareness of smoke toxicity has increased over the years, but has not yet been fully realized by all [78], [80], [81], [82]. In 2007, although 51% of surgeons considered the smoke to be harmful, residents and operating-theater nurses were more aware of its harmful effects, i.e., 78% and 91%, respectively [81]. A publication in 2020 found that although awareness had increased (95%), only 50.4% of the surgeons considered smoke very harmful and tried to avoid it (51%) [81]. Nurses also had varying degrees of awareness (44.4%–80%) [74], [81]. These rates show that there is still much room for improvement in terms of awareness of and protection against surgical smoke. In one survey [83], surgeons and anesthetists perceived less risk from the smoke and claimed lower exposure to the smoke than nurses. Males had a low perception of risk. Moreover, surgeons support a diathermy smoke-free policy less (78%) than do nurses and anesthetists. Nurses (86%) and – with a low frequency (49%) – surgeons suggest that making smoke-evacuator use mandatory is the best way to manage smoke exposure. These results show a great need for surgeons’ awareness to improve.

A review by Dixon et al. [84] contains contradictory statements about awareness. Conclusions in their abstract and in discussion sections differ. In the conclusion of the abstract, the authors stated that smoke was hazardous and advised protection. However, they claimed that high-level evidence for infectivity potential of the smoke does not exist. They seemed to accept ‘a possible link’ between the surgical smoke and HPV infection and concluded that surgical smoke does not cause permanent health problems in long-term follow-ups. Moreover, they concluded that carcinogenic chemicals had a low carcinogenic potential overall. They reviewed 28 studies, of which 92% were experimental and had a limited number of subjects. These implications drawn by those authors are beyond their study’s results. This demonstrates that even the authors’ awareness of surgical smoke is conflicted.

Stewart [85] pointed out some limitations and contradictions in a number of studies in her editorial article and concluded that the hazards of surgical smoke are generally overstated. Moreover, she claimed other authors had clear conflicts of interest in the medical device industry. The author Stewart concluded that the hazards from direct inhalation of surgical smoke is hypothetical and does not occur in the operating theater [85]. According to the author of the present article, who suffered from acute illnesses for many years due to surgical smoke, this suggestion seems more harmful than ignorance. 

In the literature, the rate of education and training on surgical smoke was reported at about 56% [3], [74], [80]. However, it may be as low as 16%–20.4% for surgeons and operating theater nurses [74], [81]. An average of 60% of healthcare professionals think that warnings and standards about the deleterious effects of surgical smoke are insufficient [78], [80]. Moreover, as the author of this article experienced, some surgeons offer no institutional support when this problem is pointed out [78]. Fortunately, despite the lack of institutional interest and support, the literature reflects that something is changing. A search in PubMed with the keyword “surgical smoke” presented 117 articles from the year 1900 up to April 2018 (in 118 years) and 215 articles up to April 2023 for the last 5 years [86], [87].

## Guidelines and regulations

The National Institute for Occupational Safety and Health (NIOSH) states five levels of actions to reduce or remove hazards in the workplace, which is named as ‘the hierarchy of controls’ [88]. NIOSH first published a guideline on controlling surgical smoke in 1996 [89]. Recommendations in the guideline are still up to date and are clearly organized in ‘the surgical smoke hierarchy of controls’ [89], [90]. 

In detail, the surgical smoke hierarchy of controls are

1. Elimination: preventing surgical smoke production and removing all the smoke emitted;
2. Substitution: evaluating alternative surgical power devices that generate less surgical smoke;
3. Engineering controls: isolating the smoke from the operating theater staff by every feasible method and device, and modifying equipment or the workspace, using protective barriers, ventilation, and more;
4. Administrative controls: establishing policies and procedures with periodical review and revision, providing education, training in work processes, ensuring adequate rest breaks, job rotation, limiting access to hazardous areas. Perioperative personnel should participate in improvement programs by relevant health-care organizations of countries;
5. Personal protective equipment: using appropriate masks, eye and face protectors, suitable surgical cap, gowns according to the power devices used, and insisting on using high quality equipment [89], [90], [91], [92].

Finally, the health care organizations of the countries are obligated to provide a surgical-smoke-free work environment, and the operating-room staff has responsibilities and obligations to protect themselves from exposure to smoke.

## Conclusion

Power devices (e.g., electrocautery, ultrasonic and laser devices) produce surgical smoke with a particle size as small as 0.007µm during the surgical procedures. Currently, more than 1,000 different chemicals have been identified in the smoke. There are high amounts of toxic, carcinogenic and mutagenic substances among these chemicals. Surgical masks cannot filter particles smaller than 5 µm. In this case, 95% of the smoke particles which crossed the mask reaches deep into the respiratory tract. 

Surgical smoke often causes headache, dizziness, nausea, eye and respiratory tract irritation, weakness, abdominal pain, dysrhythmia, hypertensive attack, etc. in the acute period. Long-term effects of surgical smoke on various diseases have consistently been ignored. 

Bacteria and viruses, mostly <2.5 µm in size, can be transported by the smoke and there is a potential risk of contamination. Among these viruses, SARS-CoV-2, HIV, HPV, HBV must be considered. 

Mutagenic and carcinogenic compounds as well as malignant cells may also be found in the smoke. Therefore, surgical smoke inhalation is a significant chemical and biological occupational hazard in the operating theater. Surgical smoke is as mutagenic as cigarette smoke. No hospital would think of allowing smoking in the operating theater to protect non-smokers. In contrast, protection from exposure to surgical smoke does not receive the analogous attention.

The quantity of the smoke changes with the technique of the surgeon, the characteristics of the power device used, the energy level set, the characteristics of the tissue processed and the ventilation or smoke evacuation system. The most effective method is to collect the smoke from the origin with an aspiration system and to evacuate it to the outside.

Awareness and legal regulations in terms of hygiene, toxicology, occupational health, and safety should be increased.

## Notes

### Competing interests

The author declares that he has no competing interest.

### Funding

None.

### Author’s ORCID

Nurettin Kahramansoy: 0000-0001-8990-5073

## Figures and Tables

**Table 1:**
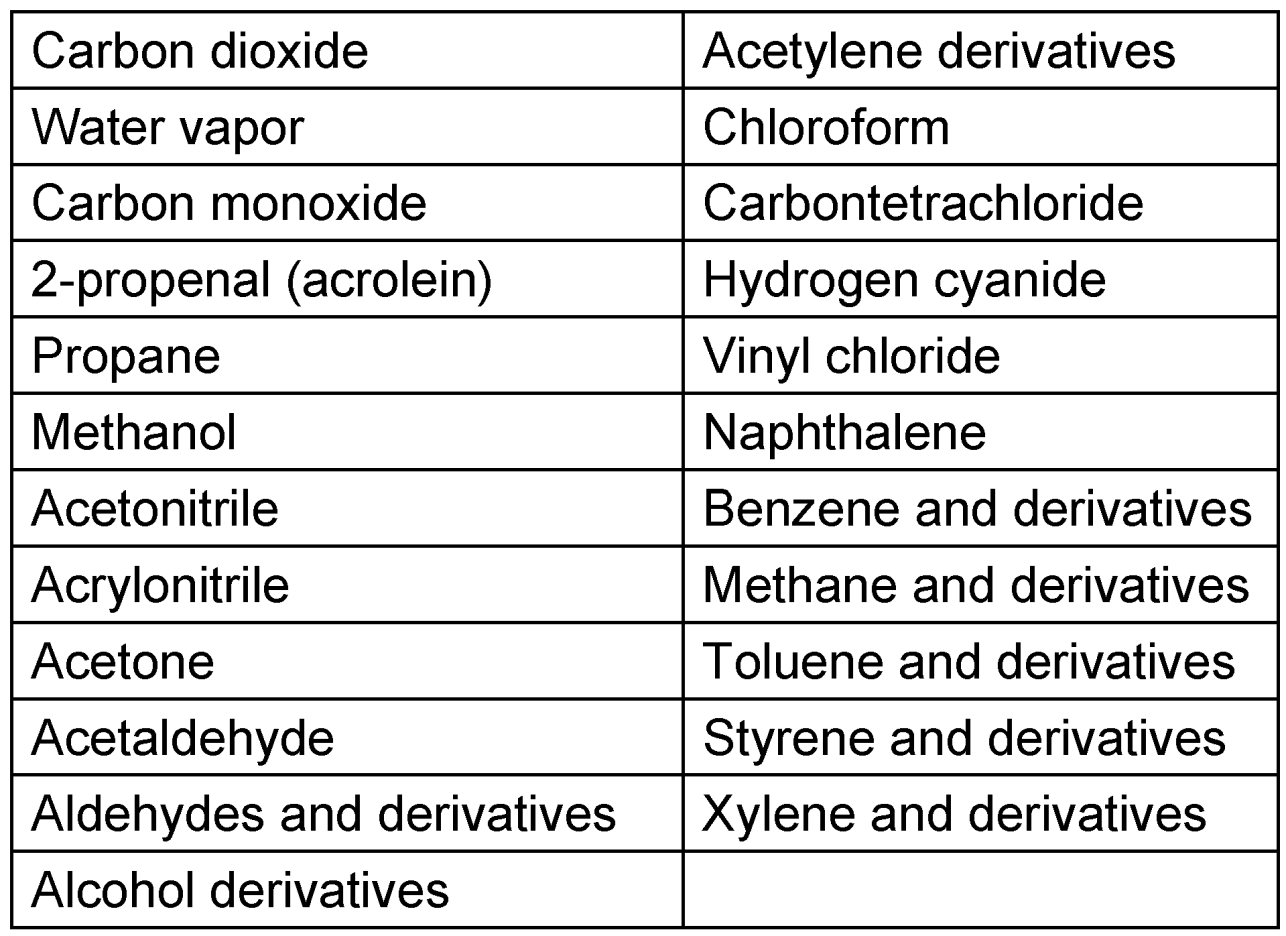
The most frequently found substances in surgical smoke (lines 1–5) in the first column) are ordered by the frequency of occurrence) [1], [9], [10], [15], [93], [94].

**Table 2:**
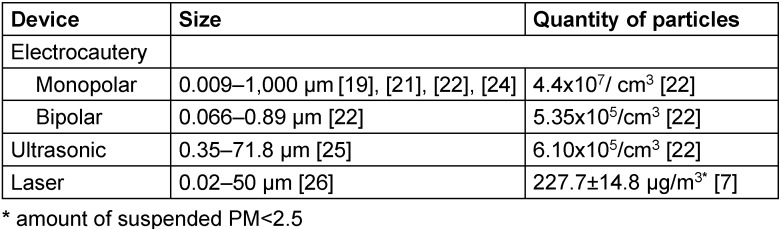
Relationship between the power devices and the size and amount of particles formed

**Table 3:**
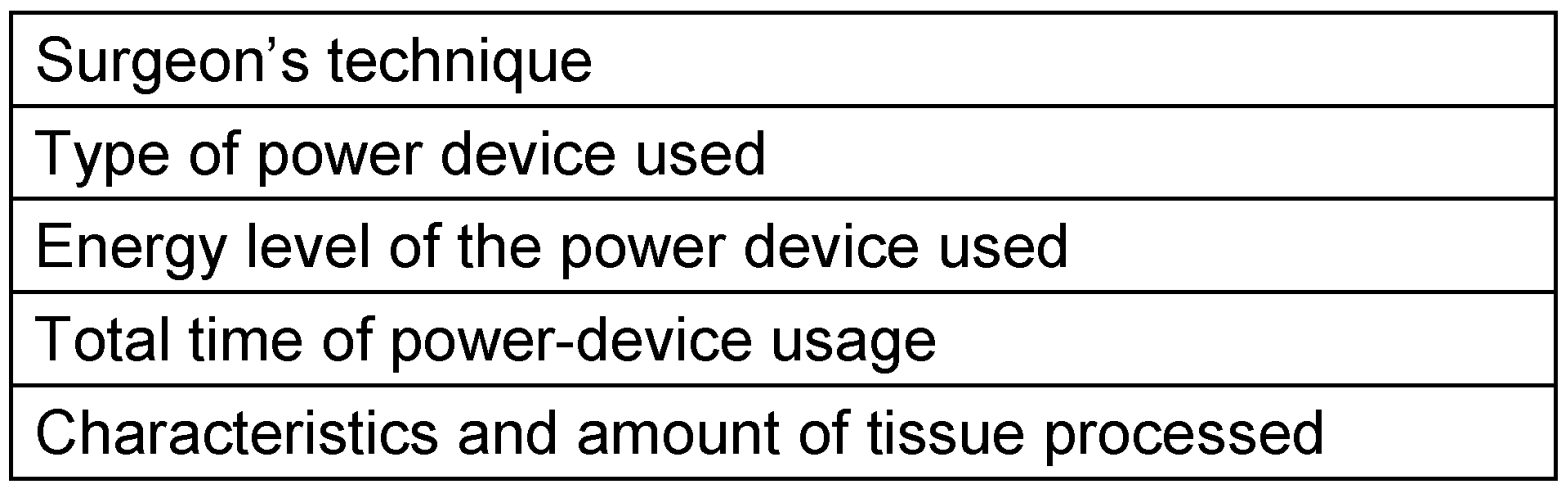
Factors affecting the amount of surgical smoke

**Table 4:**
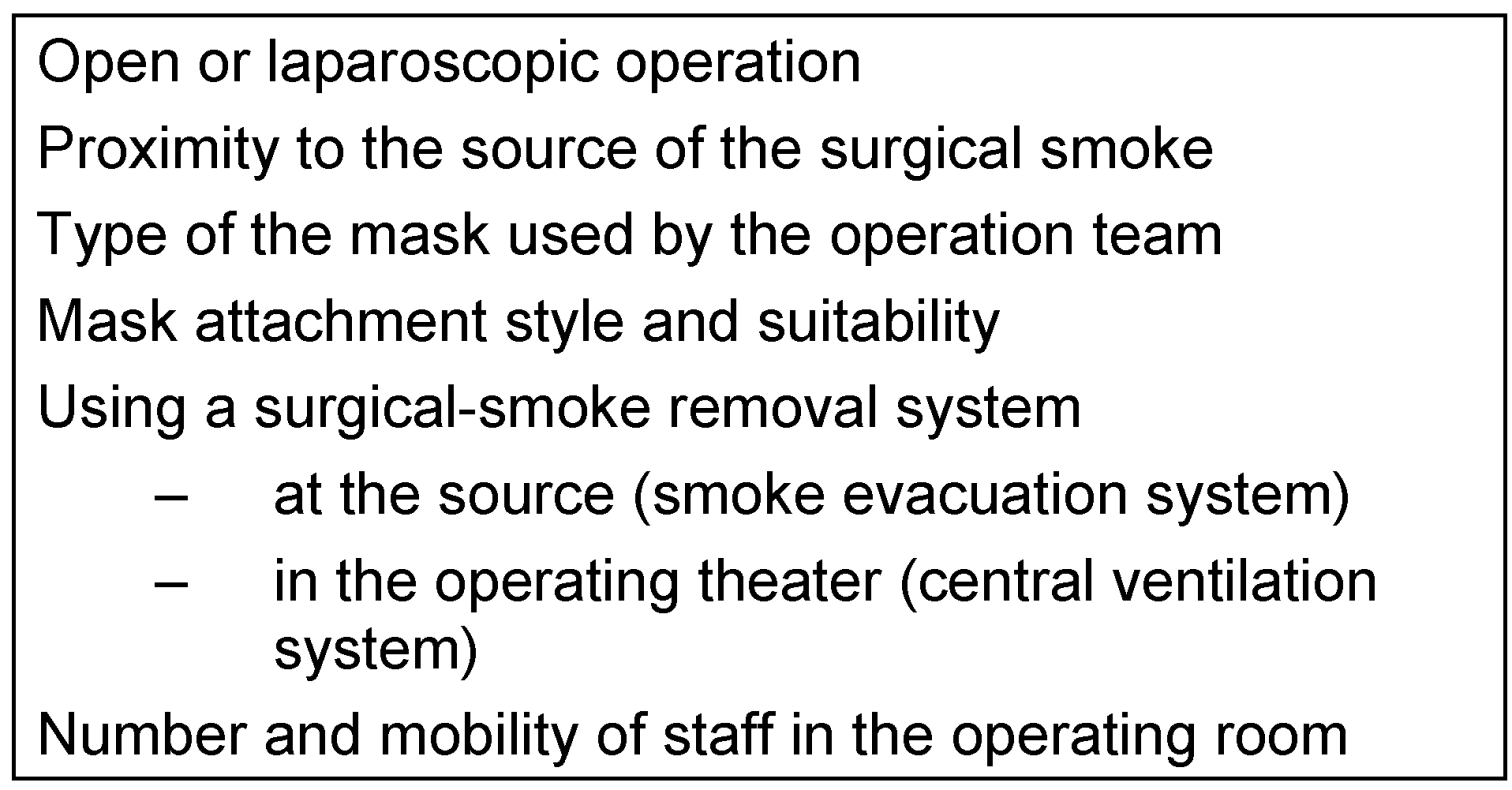
Factors affecting surgical-smoke exposure of the operating team

**Table 5:**
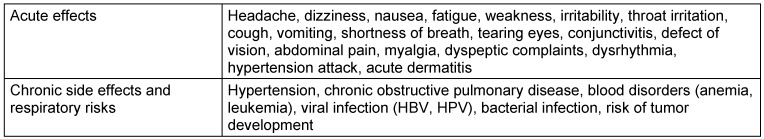
Acute and chronic clinical conditions caused by surgical smoke

## References

[1] Barrett WL, Garber SM (2003). Surgical smoke: a review of the literature. Is this just a lot of hot air?. Surg Endosc.

[2] Mowbray N, Ansell J, Warren N, Wall P, Torkington J (2013). Is surgical smoke harmful to theater staff? A systematic review. Surg Endosc.

[3] Bree K, Barnhill S, Rundell W (2017). The dangers of electrosurgical smoke to operating room personnel: a review. Workplace Health Saf.

[4] Hill DS, O'Neill JK, Powell RJ, Oliver DW (2012). Surgical smoke – a health hazard in the operating theatre: a study to quantify exposure and a survey of the use of smoke extractor systems in UK plastic surgery units. J Plast Reconstr Aesthet Surg.

[5] Fan JK, Chan FS, Chu KM (2009). Surgical smoke. Asian J Surg.

[6] Choi SH, Choi DH, Kang DH, Ha YS, Lee JN, Kim BS, Kim HT, Yoo ES, Kwon TG, Chung SK, Kim TH (2018). Activated carbon fiber filters could reduce the risk of surgical smoke exposure during laparoscopic surgery: application of volatile organic compounds. Surg Endosc.

[7] Pierce JS, Lacey SE, Lippert JF, Lopez R, Franke JE (2011). Laser-generated air contaminants from medical laser applications: a state-of-the-science review of exposure characterization, health effects, and control. J Occup Environ Hyg.

[8] Kocher GJ, Koss AR, Groessl M, Schefold JC, Luedi MM, Quapp C, Dorn P, Lutz J, Cappellin L, Hutterli M, Lopez-Hilfiker FD, Al-Hurani M, Sesia SB (2022). Electrocautery smoke exposure and efficacy of smoke evacuation systems in minimally invasive and open surgery: a prospective randomized study. Sci Rep.

[9] Morimoto G, Kawahira H, Takayama S, Lefor AK (2022). Chemical components of smoke produced from versatile training tissue models using electrocautery. Simul Healthc.

[10] Cheng MH, Chiu CH, Chen CT, Chou HH, Pao LH, Wan GH (2021). Sources and components of volatile organic compounds in breast surgery operating rooms. Ecotoxicol Environ Saf.

[11] Gianella M, Hahnloser D, Rey JM, Sigrist MW (2014). Quantitative chemical analysis of surgical smoke generated during laparoscopic surgery with a vessel-sealing device. Surg Innov.

[12] Sisler JD, Shaffer J, Soo JC, LeBouf RF, Harper M, Qian Y, Lee T (2018). In vitro toxicological evaluation of surgical smoke from human tissue. J Occup Med Toxicol.

[13] Fitzgerald JE, Malik M, Ahmed I (2012). A single-blind controlled study of electrocautery and ultrasonic scalpel smoke plumes in laparoscopic surgery. Surg Endosc.

[14] Chung YJ, Lee SK, Han SH, Zhao C, Kim MK, Park SC, Park JK (2010). Harmful gases including carcinogens produced during transurethral resection of the prostate and vaporization. Int J Urol.

[15] Ha HI, Choi MC, Jung SG, Joo WD, Lee C, Song SH, Park H (2019). Chemicals in surgical smoke and the efficiency of built-in-filter ports. JSLS.

[16] Choi C, Do IG, Song T (2018). Ultrasonic versus monopolar energy-based surgical devices in terms of surgical smoke and lateral thermal damage (ULMOST): a randomized controlled trial. Surg Endosc.

[17] Park SC, Lee SK, Han SH, Chung YJ, Park JK (2012). Comparison of harmful gases produced during GreenLight High-Performance System laser prostatectomy and transurethral resection of the prostate. Urology.

[18] International Agency for Research on Cancer (IARC) (2023). Agents classified by the IARC Monographs, Volumes 1–134.

[19] Heinsohn P, Jewett DL, Balzer L, Bennett CH, Seipel P, Rosen A (1991). Aerosols created by some surgical power tools: particle size distribution and qualitative hemoglobin content. Appl Occup Environ Hyg.

[20] Okoshi K, Kobayashi K, Kinoshita K, Tomizawa Y, Hasegawa S, Sakai Y (2015). Health risks associated with exposure to surgical smoke for surgeons and operation room personnel. Surg Today.

[21] Ragde SF, Jørgensen RB, Føreland S (2016). Characterisation of exposure to ultrafine particles from surgical smoke by use of a fast mobility particle sizer. Ann Occup Hyg.

[22] Weld KJ, Dryer S, Ames CD, Cho K, Hogan C, Lee M, Biswas P, Landman J (2007). Analysis of surgical smoke produced by various energy-based instruments and effect on laparoscopic visibility. J Endourol.

[23] Karjalainen M, Kontunen A, Saari S, Rönkkö T, Lekkala J, Roine A, Oksala N (2018). The characterization of surgical smoke from various tissues and its implications for occupational safety. PLoS One.

[24] Casey VJ, Martin C, Curtin P, Buckley K, McNamara LM (2021). Comparison of surgical smoke generated during electrosurgery with aerosolized particulates from ultrasonic and high-speed cutting. Ann Biomed Eng.

[25] Ott DE, Moss E, Martinez K (1998). Aerosol exposure from an ultrasonically activated (Harmonic) device. J Am Assoc Gynecol Laparosc.

[26] Lopez R, Lacey SE, Lippert JF, Liu LC, Esmen NA, Conroy LM (2015). Characterization of size-specific particulate matter emission rates for a simulated medical laser procedure--a pilot study. Ann Occup Hyg.

[27] In SM, Park DY, Sohn IK, Kim CH, Lim HL, Hong SA, Jung DY, Jeong SY, Han JH, Kim HJ (2015). Experimental study of the potential hazards of surgical smoke from powered instruments. Br J Surg.

[28] Ramli NA, Md Yusof NFF, Shith S, Surato A (2020). Chemical and biological compositions associated with ambient respirable particulate matter: a review. Water Air Soil Pollut.

[29] Louten J, Louten J (2016). Virus Structure and Classification.

[30] Capizzi PJ, Clay RP, Battey MJ (1998). Microbiologic activity in laser resurfacing plume and debris. Lasers Surg Med.

[31] Zhu Z, Liu N, Xia W, Liu H, Wood KB, Wang K (2021). Bacteria in surgical smoke: a self-controlled laboratory study using porcine spinal tissues. Spine (Phila Pa 1976).

[32] Carr-Locke DL, Soetikno R, Shah S, Kaltenbach T, Shergill A (2020). I Smell smoke-the must know details about the N95. Am J Gastroenterol.

[33] Kwak HD, Kim SH, Seo YS, Song KJ (2016). Detecting hepatitis B virus in surgical smoke emitted during laparoscopic surgery. Occup Environ Med.

[34] Garden JM, O'Banion MK, Shelnitz LS, Pinski KS, Bakus AD, Reichmann ME, Sundberg JP (1988). Papillomavirus in the vapor of carbon dioxide laser-treated verrucae. JAMA.

[35] Neumann K, Cavalar M, Rody A, Friemert L, Beyer DA (2018). Is surgical plume developing during routine LEEPs contaminated with high-risk HPV? A pilot series of experiments. Arch Gynecol Obstet.

[36] Yokoe T, Kita M, Odaka T, Fujisawa J, Hisamatsu Y, Okada H (2021). Detection of human coronavirus RNA in surgical smoke generated by surgical devices. J Hosp Infect.

[37] Baggish MS, Poiesz BJ, Joret D, Williamson P, Refai A (1991). Presence of human immunodeficiency virus DNA in laser smoke. Lasers Surg Med.

[38] Brüske-Hohlfeld I, Preissler G, Jauch KW, Pitz M, Nowak D, Peters A, Wichmann HE (2008). Surgical smoke and ultrafine particles. J Occup Med Toxicol.

[39] Wang HK, Mo F, Ma CG, Dai B, Shi GH, Zhu Y, Zhang HL, Ye DW (2015). Evaluation of fine particles in surgical smoke from an urologist's operating room by time and by distance. Int Urol Nephrol.

[40] Okoshi K, Hida K, Kinoshita K, Morishima T, Nagai Y, Tomizawa Y, Yorozuya K, Nishida T, Matsumoto H, Yamato H (2022). Measurement of particulate matter 2.5 in surgical smoke and its health hazards. Surg Today.

[41] Kameyama H, Otani T, Yamazaki T, Iwaya A, Uehara H, Harada R, Hirai M, Komatsu M, Kubota A, Katada T, Kobayashi K, Sato D, Yokoyama N, Kuwabara S, Tanaka Y, Sawakami K (2022). Comparison of surgical smoke between open surgery and laparoscopic surgery for colorectal disease in the COVID-19 era. Surg Endosc.

[42] Smith JP, Topmiller JL, Shulman S (1990). Factors affecting emission collection by surgical smoke evacuators. Lasers Surg Med.

[43] Stewart CL, Raoof M, Lingeman R, Malkas L, Flores V, Caldwell K, Fong Y, Melstrom K (2021). A Quantitative analysis of surgical smoke exposure as an occupational hazard. Ann Surg.

[44] Shah NR (2012). Commentary On: "Surgical Smoke - A Health Hazard in the Operating Theatre: A Study to Quantify Exposure and a Survey of the Use of Smoke Extractor Systems in UK Plastic Surgery Units". Ann Med Surg (Lond).

[45] Mintz Y, Arezzo A, Boni L, Baldari L, Cassinotti E, Brodie R, Uranues S, Zheng M, Fingerhut A (2020). The risk of COVID-19 transmission by laparoscopic smoke may be lower than for laparotomy: a narrative review. Surg Endosc.

[46] Assadian O, Golling M, Krüger CM, Leaper D, Mutters NT, Roth B, Kramer A (2021). Surgical site infections: guidance for elective surgery during the SARS-CoV-2 pandemic - international recommendations and clinical experience. J Hosp Infect.

[47] Vigneswaran Y, Prachand VN, Posner MC, Matthews JB, Hussain M (2020). What Is the Appropriate Use of Laparoscopy over Open Procedures in the Current COVID-19 Climate?. J Gastrointest Surg.

[48] (2019). DIN EN ISO 29463-2:2019-05: High-efficiency filters and filter media for removing particles in air - Part 2: Aerosol production, measuring equipment and particle-counting statistics.

[49] Romano F, Milani S, Gustén J, Joppolo CM (2020). Surgical smoke and airborne microbial contamination in operating theatres: influence of ventilation and surgical phases. Int J Environ Res Public Health.

[50] Tokuda Y, Okamura T, Maruta M, Orita M, Noguchi M, Suzuki T, Matsuki H (2020). Prospective randomized study evaluating the usefulness of a surgical smoke evacuation system in operating rooms for breast surgery. J Occup Med Toxicol.

[51] Moon HJ, Lee WJ (2022). Measurement and control of surgical smoke to enhance surgical team safety. J Korean Med Sci.

[52] Liu Y, Song Y, Hu X, Yan L, Zhu X (2019). Awareness of surgical smoke hazards and enhancement of surgical smoke prevention among the gynecologists. J Cancer.

[53] Van Gestel EAF, Linssen ES, Creta M, Poels K, Godderis L, Weyler JJ, De Schryver A, Vanoirbeek JAJ (2020). Assessment of the absorbed dose after exposure to surgical smoke in an operating room. Toxicol Lett.

[54] Weber A, Willeke K, Marchioni R, Myojo T, McKay R, Donnelly J, Liebhaber F (1993). Aerosol penetration and leakage characteristics of masks used in the health care industry. Am J Infect Control.

[55] Gao S, Koehler RH, Yermakov M, Grinshpun SA (2016). Performance of facepiece respirators and surgical masks against surgical smoke: simulated workplace protection factor study. Ann Occup Hyg.

[56] Douglas JDM, McLean N, Horsley C, Higgins G, Douglas CM, Robertson E (2020). COVID-19: smoke testing of surgical mask and respirators. Occup Med (Lond).

[57] Benson SM, Novak DA, Ogg MJ (2013). Proper use of surgical n95 respirators and surgical masks in the OR. AORN J.

[58] Chan JF, Yuan S, Zhang AJ, Poon VK, Chan CC, Lee AC, Fan Z, Li C, Liang R, Cao J, Tang K, Luo C, Cheng VC, Cai JP, Chu H, Chan KH, To KK, Sridhar S, Yuen KY (2020). Surgical mask partition reduces the risk of noncontact transmission in a golden syrian hamster model for coronavirus disease 2019 (COVID-19). Clin Infect Dis.

[59] Lee T, Soo JC, LeBouf RF, Burns D, Schwegler-Berry D, Kashon M, Bowers J, Harper M (2018). Surgical smoke control with local exhaust ventilation: Experimental study. J Occup Environ Hyg.

[60] Ekci B (2020). Easy-to-use electrocautery smoke evacuation device for open surgery under the risk of the COVID-19 pandemic. J Int Med Res.

[61] Ouzzane A, Colin P (2020). Cost-effective filtrating suction to evacuate surgical smoke in laparoscopic and robotic surgery during the COVID-19 pandemic. Surg Laparosc Endosc Percutan Tech.

[62] Andrade WP, Gonçalves GG, Medeiros LC, Araujo DCM, Pereira GTG, Moraes DMP, Spencer RMSSB (2020). Low-cost, safe, and effective smoke evacuation device for surgical procedures in the COVID-19 age. J Surg Oncol.

[63] Hahn KY, Kang DW, Azman ZAM, Kim SY, Kim SH (2017). Removal of hazardous surgical smoke using a built-in-filter trocar: a study in laparoscopic rectal resection. Surg Laparosc Endosc Percutan Tech.

[64] Takahashi H, Yamasaki M, Hirota M, Miyazaki Y, Moon JH, Souma Y, Mori M, Doki Y, Nakajima K (2013). Automatic smoke evacuation in laparoscopic surgery: a simplified method for objective evaluation. Surg Endosc.

[65] Liang JH, Xu CL, Wang LH, Hou JG, Gao XF, Sun YH (2008). Irrigation eliminates smoke formation in laser laparoscopic surgery: ex vivo results. Surg Laparosc Endosc Percutan Tech.

[66] Ansell J, Warren N, Wall P, Cocks K, Goddard S, Whiston R, Stechman M, Scott-Coombes D, Torkington J (2014). Electrostatic precipitation is a novel way of maintaining visual field clarity during laparoscopic surgery: a prospective double-blind randomized controlled pilot study. Surg Endosc.

[67] Theodore C, Simpson GS, Walsh CJ (2021). Theatre ventilation. Ann R Coll Surg Engl.

[68] Hofer V, Kriegel M (2022). Exposure of operating room surgical staff to surgical smoke under different ventilation schemes. Indoor Air.

[69] Popp W, Alefelder C, Bauer S, Daeschlein G, Geistberger P, Gleich S, Herr C, Hübner NO, Jatzwauk L, Kohnen W, Külpmann R, Lemm F, Loczenski B, Spors J, Walger P, Wehrl M, Zastrow KD, Exner M (2019). Air quality in the operating room: Surgical site infections, HVAC systems and discipline - position paper of the German Society of Hospital Hygiene (DGKH). GMS Hyg Infect Control.

[70] Külpmann R, Christiansen B, Kramer A, Lüderitz P, Pitten FA, Wille F, Zastrow KD, Lemm F, Sommer R, Halabi M (2016). Hygiene guideline for the planning, installation, and operation of ventilation and air-conditioning systems in health-care settings - Guideline of the German Society for Hospital Hygiene (DGKH). GMS Hyg Infect Control.

[71] Newsom RB, Amara A, Hicks A, Quint M, Pattison C, Bzdek BR, Burridge J, Krawczyk C, Dinsmore J, Conway J (2021). Comparison of droplet spread in standard and laminar flow operating theatres: SPRAY study group. J Hosp Infect.

[72] Pokrywka M, Byers K (2013). Traffic in the operating room: a review of factors influencing air flow and surgical wound contamination. Infect Disord Drug Targets.

[73] Pasquarella C, Balocco C, Colucci ME, Saccani E, Paroni S, Albertini L, Vitali P, Albertini R (2020). The influence of surgical staff behavior on air quality in a conventionally ventilated operating theatre during a simulated arthroplasty: a case study at the university hospital of Parma. Int J Environ Res Public Health.

[74] Ünver S, Topçu SY, Fındık ÜY (2016). Surgical smoke, me and my circle. Int J Caring Sci.

[75] Okgün Alcan A, Yavuz Van Giersbergen M, Tanıl V, Dinçarslan G, Hepçivici Z, Kurcan Ç, Arıkan E, Dere T (2017). Bir ünIversIte hastanesInde cerrahI duman rIsklerI ve koruyucu önlemlerIn IncelenmesI. Ege Üniversitesi Hemsirelik Fakültesi Dergisi.

[76] Yu CL, Hsieh SI, Lin LH, Chi SF, Huang TH, Yeh SL, Wang C (2022). Factors associated with surgical smoke self-protection behavior of operating room nurses. Healthcare (Basel).

[77] Iwai K, Mizuno S, Miyasaka Y, Mori T (2005). Correlation between suspended particles in the environmental air and causes of disease among inhabitants: cross-sectional studies using the vital statistics and air pollution data in Japan. Environ Res.

[78] Spearman J, Tsavellas G, Nichols P (2007). Current attitudes and practices towards diathermy smoke. Ann R Coll Surg Engl.

[79] Padmanabhan AK, Prabhuji MLV, Mampuzha S, Subramanya AP (2020). Surgical smoke in dental practice: a potential biohazard. Dentist Case Rep.

[80] Steege AL, Boiano JM, Sweeney MH (2016). Secondhand smoke in the operating room? Precautionary practices lacking for surgical smoke. Am J Ind Med.

[81] Michaelis M, Hofmann FM, Nienhaus A, Eickmann U (2020). Surgical smoke-hazard perceptions and protective measures in german operating rooms. Int J Environ Res Public Health.

[82] Ilce A, Yuzden GE, Yavuz van Giersbergen M (2017). The examination of problems experienced by nurses and doctors associated with exposure to surgical smoke and the necessary precautions. J Clin Nurs.

[83] Barber L, Lane R, Holmes L, Murray N, Hamill JK (2022). Surgical smoke: how an issue in healthcare fits a planetary health framework. N Z Med J.

[84] Dixon K, Dasgupta P, Vasdev N (2023). A systematic review of the harmful effects of surgical smoke inhalation on operating room personnel. Health Sci Rev.

[85] Stewart CL (2022). Do we need to reduce surgical smoke?. Ann Transl Med.

[86] PubMed (1990–2018). “surgical smoke”.

[87] PubMed (2018–2023). “surgical smoke”.

[88] National Institute for Occupational Safety and Health (2023). Hierarchy of Controls.

[89] Centers for Diseas Control and Prevention (CDC), National Institute for Occupational Safety and Health National Institute for Occupational Safety and Health (NIOSH) (2014). Control of smoke from laser/electric surgical procedures. DHHS (NIOSH) Publication No. 96-128, (HC 11), 1996.

[90] Williams K (2022). Guidelines in Practice: Surgical Smoke Safety. AORN J.

[91] Canadian Centre for Occupational Health and Safety (CCOHS) (2023). Laser plumes – Health Care.

[92] Ogg MJ, et al. (2017). Patient and worker safety: Guideline for Surgical Smoke Safety AORN Guidelines for Perioperative Practice.

[93] Dobrogowski M, Wesołowski W, Kucharska M, Sapota A, Pomorski LS (2014). Chemical composition of surgical smoke formed in the abdominal cavity during laparoscopic cholecystectomy--assessment of the risk to the patient. Int J Occup Med Environ Health.

[94] Claudio CV, Ribeiro RP, Martins JT, Marziale MH, Solci MC, Dalmas JC (2017). Polycyclic aromatic hydrocarbons produced by electrocautery smoke and the use of personal protective equipment 1. Rev Lat Am Enfermagem.

